# Draft Genome Sequence of *Pseudomonas aeruginosa* SG17M, an Environmental Isolate Belonging to Clone C, Prevalent in Patients and Aquatic Habitats

**DOI:** 10.1128/genomeA.00186-14

**Published:** 2014-03-20

**Authors:** Changhan Lee, Verena Peters, Öjar Melefors, Ute Römling

**Affiliations:** a Department of Microbiology, Tumor and Cell Biology, Karolinska Institutet, Stockholm, Sweden; b Department of Microbiology, Public Health Agency of Sweden, Solna, Sweden

## Abstract

*Pseudomonas aeruginosa* SG17M is an environmental isolate recovered from river water in the city of Mulheim, Germany. SG17M belongs to clone C, which is distributed worldwide. This is the first clone C strain whose genome sequence has been determined.

## GENOME ANNOUNCEMENT

*Pseudomonas aeruginosa* is a paradigm of a highly successful nosocomial pathogen capable of causing a wide variety of infections, like those in wounds, ears, eyes, and the urinary tract (1). This opportunistic pathogen also causes chronic infections in the cystic fibrosis (CF) lung, affecting patient morbidity and mortality (2). Besides its clinical impact, *P. aeruginosa* is widely distributed in diverse environmental and technical niches, such as the aquatic habitats covering natural and processed water (3, 4). However, the mechanisms of how *P. aeruginosa* survives in the environment and successfully infects patients and how *P. aeruginosa* adapts to these various environmental conditions have not been entirely elucidated. One of the most abundant groups of closely related *P. aeruginosa* strains is clone C, which is distributed worldwide in patients, covering a spectrum of diseases, and is prevalent in the aquatic environment (5–11). In this report, we have determined the whole-genome sequence of *P. aeruginosa* SG17M, a clone C strain isolated from a river in Germany (7). We anticipate that the analysis of this genome sequence will be a basis for unraveling the unique features of clone C strains that leads to their prevalence in the environment and in human infections.

The genomic DNA of *P. aeruginosa* SG17M was extracted using the GenElute bacterial genomic DNA kit (catalog no. NA2110; Sigma). The genome was sequenced by 454 sequencing (in-house) and by using an Illumina HiSeq 2000 sequencing platform at the Beijing Genomics Institute (BGI)-Hong Kong (http://www.genomics.cn/). Based on two whole-genome shotgun libraries, 755 Mb of data was produced by the 500-bp library and 377 Mb of data was produced by the 6,000-bp library. The resultant sequences were assembled using SOAP*denovo* software (version 2.04) (http://soap.genomics.org.cn/). Based on the assembly result of SG17M, we found the genome size to be 6.87 Mb, the G+C content to be 66.19%, the number of scaffolds to be 17, and the number of contigs to be 319. Genomic analysis of the SG17M genome using Kyoto Encyclopedia of Gene and Genomes (KEGG) annotation (http://www.genome.jp/tools/kaas/) found that the genome contains 6,521 genes; the total length of the genes is 6,138,813 bp, which makes up 89.05% of the total genome size.

The genome structure of clone C strains has been extensively characterized by low-resolution methods, such as pulsed-field gel electrophoresis, whereby chromosomal plasticity has been observed between clone C strains created by insertions, deletions, the rearrangement of genomic islands and islets, and large chromosomal inversions (12). Although some selected genomic islands of SG17M and other clone C strains, including a common 102-kbp plasmid, have been sequenced (13–15), the whole-genome sequence of the SG17M *P. aeruginosa* clone C strain reported here will provide the basis for elucidating the genetic diversity among clone C strains, to define the pangenome of *P. aeruginosa* clone C, and to further investigate in detail the success of clone C strains in host infection, environmental survival, and transmission throughout the world. Furthermore, the genome sequence will also help to understand the specific physiological features of highly successful strains in general, and to identify novel antimicrobial targets that will contribute to develop next-generation antimicrobial agents.

### Nucleotide sequence accession numbers.

This whole-genome shotgun project has been deposited at DDBJ/EMBL/GenBank under the accession no. JALF00000000. The version described in this paper is version JALF01000000.
